# Suicide attempt-related emergency department visits among adolescents: a nationwide population-based study in Korea, 2016–2019

**DOI:** 10.1186/s12888-022-04043-6

**Published:** 2022-06-22

**Authors:** Kyung-Shin Lee, Daesung Lim, Jong-Woo Paik, Youn Young Choi, Jaehyun Jeon, Ho Kyung Sung

**Affiliations:** 1 Research Institute for Public Health, National Medical Center, Seoul, Korea; 2 Department of Emergency Medicine, Gyeongsang National University College of Medicine, Gyeongsang National University Changwon Hospital, Changwon, Korea; 3 Department of Psychiatry, Kyung Hee University Medical Center, Seoul, Korea; 4 Department of Pediatrics, National Medical Center, Seoul, Korea; 5 Division of Infectious Diseases, National Medical Center, Seoul, Korea; 6 National Emergency Medical Center, National Medical Center, 245 Eulgi-ro, Jung-gu, 04564 Seoul, Korea

**Keywords:** Suicide attempt, Adolescence, Emergency department, Annual percentage change

## Abstract

**Background:**

The incidence of adolescent suicide in Korea is increasing; however, nationwide data regarding short-term prediction of suicide attempts (SAs) is still limited. Therefore, this study aimed to investigate the incidence of SA-related annual emergency department (ED) visits among adolescents in Korea from 2016 to 2019 and to summarize the corresponding demographic and clinical characteristics based on the dispositions of SA-related ED visits.

**Methods:**

Most referral hospitals provide relevant essential ED information to the National Emergency Medical Center through the National Emergency Department Information System (NEDIS). We analyzed NEDIS data on adolescent visits (aged < 20 years) for a 4-year period from 2016 to 2019. Patients were classified into the discharge and hospitalization groups for comparison, and jointpoint regression analysis was used to identify the years in which there was a change in annual percentage change (APC) in age- and sex-standardized incidence rates of SA-related ED visits. The characteristics of patients in the discharge group and hospitalization group subgroups were also compared.

**Results:**

The APC in the incidence rate of SA-related ED visits in the 4-year study period revealed a 35.61% increase. The incidence rate increase was higher among females (APC: 46.26%) than among males (APC: 17.95%). Moreover, the incidence rate increased faster in mid-adolescence patients (APC: 51.12%) than in late-adolescence patients (APC: 26.98%). The proportion of poisoning as the SA method was 69.7% in the hospitalization group and 34.5% in the discharge group (*p* < 0.001).

**Discussion:**

Our findings suggest that an increase in the number of SA-related ED visits among female and mid-adolescent patients represented the largest increase in SA-related ED visits from 2016 to 2019. Accordingly, evidence-based suicide prevention programs need to be customized based on sex and age, and further diversification of health care systems is needed through analysis of the characteristics of the dispositions of SA-related ED visits.

**Supplementary Information:**

The online version contains supplementary material available at 10.1186/s12888-022-04043-6.

## Background

Suicide in adolescents is a critical public health issue worldwide [1]. A recent World Health Organization report revealed that suicide represents the third leading cause of death among adolescents aged 14–19 years, after road injury and interpersonal violence [2]. Although the suicide rate in adolescents is reported to be stable or declining in many developed countries over the past two decades with the introduction of various prevention programs, it is still on the rise in some countries, such as the United States (US), United Kingdom, Japan, and Korea [3–7].

Data from the World Mental Health Survey indicate that adolescence is a period when the risk of initial onset of suicide ideation increases rapidly and that 29% of such ideators actually do attempt suicide [8]. Furthermore, a growing body of evidence indicates that suicide attempts (SAs) are associated with an increased risk of repeated attempts and subsequent death [9–13]. The risk of repeated SAs by adolescents who have made the first SA varies according to region but can be as high as 27% [11], and in the first year after a SA, up to 2% of attempters die by suicide [14–16]. A US cohort study on 813 adolescents with a history of SA reported a suicide rate of 3.6% by the age of 31 years [17]. In fact, it is necessary to understand the magnitude and characteristics associated with adolescent SA in order to guide suicide prevention and intervention efforts for all age groups and not just adolescents [18].

Emergency departments (EDs) often serve as the first contact point of medical care for adolescents who attempt suicide [19, 20]. EDs can be part of a national or regional surveillance system for monitoring SAs, but only a limited number of studies have been conducted due to data availability issues [18]. In addition, EDs evaluate the physical injuries of suicide attempters and serve as regional referral centers for comorbid mental health conditions [21]. However, little is known about the role of EDs in providing SA care in several developed countries where adolescent suicide rates are reported to be increasing. Therefore, this study aimed to investigate the changes in adolescent SA-related ED visits over time as well as the corresponding demographic and clinical characteristics using nationwide data in Korea.

## Methods

### Study design and data sources

This nationwide population-based study included all adolescents (aged 14–19 years) with SA-related ED visits from 2016 to 2019. An SA-related ED visit was defined as an ED visit for injury due to intentional self-harm according to the Columbia Classification Algorithm of Suicide Assessment [22]. The data of these patients were extracted from the National Emergency Department Information System (NEDIS) database. The NEDIS was established in 2003 based on the Emergency Medical Service Act to evaluate the quality of care provided nationwide in EDs in Korea. The NEDIS prospectively collects data from each ED visit, including information regarding demographics (age, sex, postal code address, and insurance status), route of visit (direct or transferred), mode of arrival to ED (emergency medical service [EMS] use or other), arrival and discharge times, initial triage result in terms of the Korean Triage and Acuity Scale (KTAS) score, and the corresponding intentionality and mechanism, disposition, and diagnosis codes based on the International Classification of Disease 10th Edition (ICD-10). For each visit, corresponding data regarding hospital characteristics, including total staffed beds, ED level, and urban-rural location, were also obtained. All patient-related information was anonymized according to the protocol (https://www.e-gen.or.kr/english/). During the study period from 2016 to 2019, the participation rate of nationwide EDs in the NEDIS was 408 of 413 (98.8%) EDs in 2016, 413 of 416 (99.3%) EDs in 2017, 399 of 401 (99.5%) EDs in 2018, and 401 of 402 (99.8%) EDs in 2019. The detailed design and variables of the NEDIS database have been described elsewhere [23–25].

### Measurements

The outcome of interest was the number of annual SA-related ED visits among adolescents from 2016 to 2019. We also examined trends in SA-related ED visits during this period.

### Statistical analysis

The annual age- and sex-standardized incidence rates of SA-related ED visits among adolescents were calculated using the direct standardization method, with the 2020 Korean population obtained from Statistics Korea (https://kostat.go.kr/) as the standard population. To examine the trends in the age- and sex-standardized incidence rates across the study period, joinpoint regression analysis was performed, as described previously [26]. We used the Monte Carlo permutation method to select the least number of linear segments in which additional joinpoints did not add statistically significant linear trends [27]. The annual percentage change (APC) was transformed from the slope coefficient of each regression line. We also calculated APCs for groups stratified according to age and sex.

The demographic and clinical characteristics of adolescents with SA-related ED visits are presented as frequencies and percentages and were compared using the chi-square test according to the ED disposition (discharge vs. hospitalization groups). Patients were divided into the discharge and hospitalized groups for comparisons. Furthermore, we performed sensitivity analysis to compare subgroup characteristics in the discharge (discharged against medical advice [AMA], discharged to home, and others) and hospitalized (general ward [GW] admission and intensive care unit [ICU] admission) groups. We also determined the top 10 most common for primary diagnosis by the type of disposition.

All data preparation and statistical analysis were performed using SAS version 9.4 (SAS Institute, Cary, NC) and R version 4.1.1. (R Development Core Team, https://cran.r-project.org/), except for joinpoint regression analysis, which was conducted using the Joinpoint Trend Analysis Software, version 4.8.0.1 [28]. Statistical significance was set at *p* < 0.05, and a two-sided test was used.

### Ethics

 This study was approved by the Institutional Review Board of the National Medical Center (approval number NMC-2021-10-123) and conformed to the provisions of the Declaration of Helsinki. The requirement for obtaining informed consent from patients was waived by the board due to the observational nature of the study.

## Results

### Trends in SA-related ED visits

**Fig. 1:**
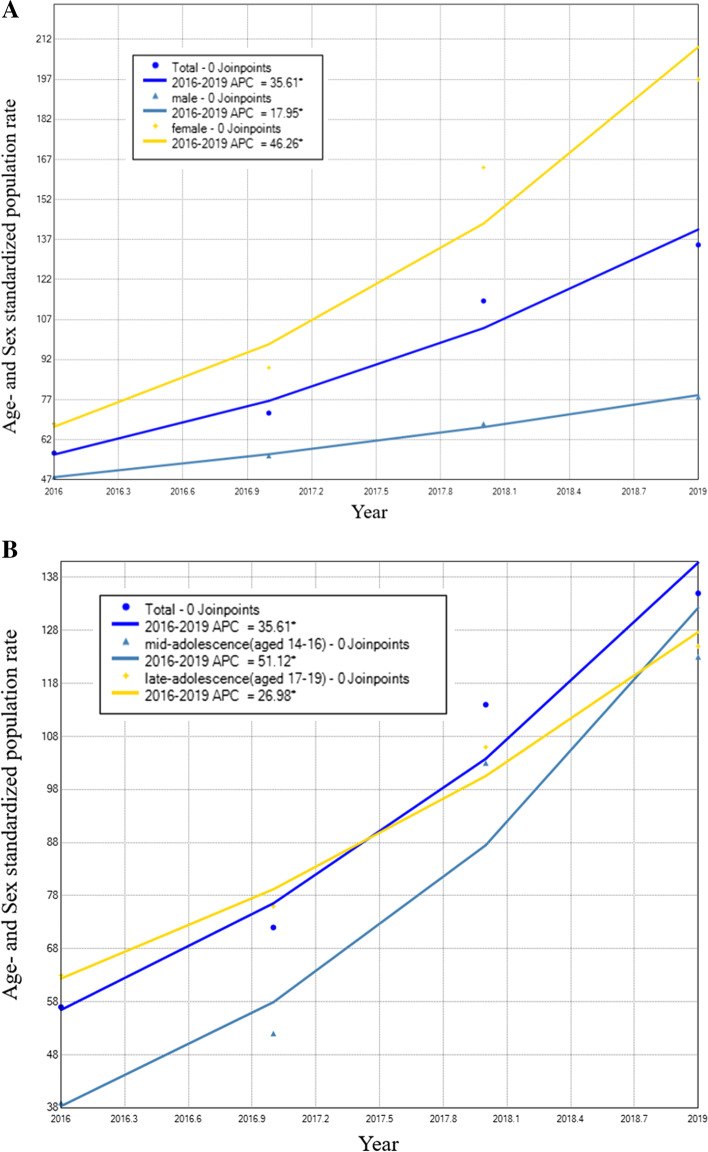
Trends in age- and sex-standardized incidence rates of SA-related ED visits among adolescents from 2016 to 2019 by sex and age

Figure S1 shows the sex- and age-standardized incidence rate of SA-related ED visits among adolescents from 2016 to 2019, which increased from 57.5/10^5^ to 2016 to 135.5/10^5^ in 2019. The APC increased by 35.61% overall and by 17.95% among males and 46.26% among females (*p* < 0.001) (Fig. 1A). Moreover, the APC increase was steeper in the mid-adolescence patients (aged 14–16 years) than that in the late-adolescence patients (aged 17–19 years) (APC, 51.12% vs. 26.98%; *p* < 0.001) (Fig. 1B).

Figure S2 shows the ED disposition among SA-related ED visits from 2016 to 2019. The discharge group had the highest proportion of SA-related ED visits compared with the disposition group (from 66.7% to 2016 to 71.4% in 2019). The proportions in the GW admission and death subgroups decreased from 2016 to 2019 (17.9% vs. 14.8% and 1.9% vs. 1.1%, respectively). The proportions in the transfer and ICU admission subgroups also changed slightly from 2016 to 2019 (4.0% vs. 5.0% and 9.3% vs. 8.7%, respectively).

### Comparison between the discharge and hospitalization groups and corresponding subgroups

**Fig. 2:**
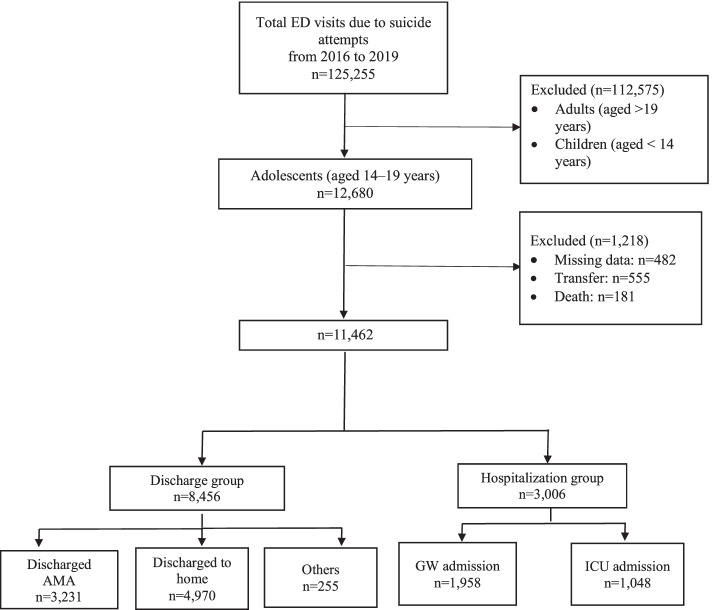
Participant selection flowchart

From 2016 to 2019, the total number of SA-related ED visits was 125,255. To estimate the demographic and clinical characteristics of adolescents with SA-related ED visits compared between the discharge group and hospitalization groups, we excluded 112,575 including adults (aged > 20 years) and children (aged < 14 years), and we also 482 patients with missing data, 555 patients in the transfer group, and 181 patients in the death group. Thus, 11,462 adolescents were included in the final analysis (Fig. 2).

All the patients were classified into the discharge (*n* = 8,456) or hospitalization group (*n* = 3,006) based on the type of disposition. In the discharge group, there were 3,231 patients in the AMA subgroup, 4,970 in the discharged to home subgroup, and 255 in the others subgroup. In the hospitalization group, there were 1,958 patients in the GW admission subgroup and 1,048 patients in the ICU admission subgroup.

Table 1 presents the overall demographic and clinical characteristics of patients in the discharge and hospitalization groups. There were significant differences in patient age group, sex, EMS use, KTAS score, year of ED visit, methods of SA, major treatment subject, time from event to arrival, time of arrival, number of hospital beds, and length of stay between the two groups. Conversely, no significant differences were noted in insurance type, season at ED visit, and ED region between the discharge and hospitalization groups. Interestingly, the data showed that the most common method of SA was poisoning, especially in the hospitalization group (69.7%, which was significantly higher than that in the discharge group; *p* < 0.0001). However, the most common method of SA in the discharge group was cutting and piercing (48.2%). Overall, > 50% of patients with SA-related ED visits reported over 600 beds in the hospital where they were hospitalized. The proportion of > 6 h length of stay was higher in the hospitalization group than in the discharge group (31.9% vs. 19.6%, respectively).

**Table 1:** Demographic and clinical characteristics of adolescent patients with suicide attempt-related ED visits from 2016 to 2019

Variable	Total (*n *= 11,462)	Discharge group (*n* = 8,456)	Hospitalization group (*n* = 3,006)	*p* value
	n(%)	n(%)	n(%)	
Age				
14–16 years	4,317 (37.6)	3,066 (36.3)	1,251 (41.6)	< 0.0001
17–19 years	7,145 (62.3)	5,390 (63.7)	1,755 (58.3)	
Female sex	7,476 (65.2)	5,357 (63.3)	2,119 (70.4)	< 0.0001
Insurance type				
NHI	9,213 (80.4)	6,840 (80.8)	2,373 (78.9)	0.069
Medicaid	1,005 (8.8)	721 (8.5)	284 (9.5)	
Other	1,244 (10.9)	895 (10.5)	349 (11.6)	
EMS use	4,669 (40.7)	3,337 (39.4)	1,332 (44.3)	< 0.0001
KTAS score				
1–3	6,390 (55.7)	3,987 (47.1)	2,403 (79.9)	< 0.0001
4–5	5,072 (44.2)	4,469 (52.8)	603 (20.0)	
Year				
2016	1,894 (16.5)	1,344 (15.8)	550 (18.3)	0.011
2017	2,266 (19.7)	1,677 (19.8)	589 (19.5)	
2018	3,410 (29.7)	2,514 (29.7)	896 (29.8)	
2019	3,892 (33.9)	2,921 (34.5)	971 (32.3)	
Season				
Spring	2,760 (24.0)	2,028 (24.0)	732 (24.3)	0.774
Summer	3,263 (28.4)	2,401 (28.4)	862 (28.6)	
Fall	3,115 (27.1)	2,320 (27.4)	795 (26.4)	
Winter	2,324 (20.2)	1,707 (20.2)	617 (20.5)	
Method of suicide attempts				
Poisoning	5,009 (43.7)	2,916 (34.5)	2,093 (69.7)	< 0.0001
Cutting and piercing	4,507 (39.4)	4,070 (48.2)	437 (14.5)	
Others	1,937 (16.9)	1,464 (17.3)	473 (15.7)	
Major treatment subject				
Psychiatry	2,076 (18.1)	1,715 (20.3)	361 (12.0)	< 0.0001
Time from event to arrival (h)	1.1 (0.6–3.9)	1.0 (0.6–3.0)	2.0 (0.7–6.0)	< 0.0001
Time of arrival				
07:00–14:59	2,693 (23.5)	1,822 (21.5)	871 (28.9)	< 0.0001
15:00–22:59	4,414 (38.5)	3,236 (38.2)	1,178 (39.1)	
23:00–06:59	4,355 (38.0)	3,398 (40.1)	957 (31.8)	
Region				
Urban	5,379 (46.9)	4,014 (47.5)	1,365 (45.4)	0.051
Rural	6,083 (53.0)	4,442 (52.5)	1,641 (54.5)	
Hospital beds				
≥ 600	5,508 (48.0)	3,996 (47.2)	1,512 (50.3)	0.004
300–599	1,433 (12.5)	1,050 (12.4)	383 (12.7)	
< 300	4,521 (39.4)	3,410 (40.3)	1,111 (36.9)	
Length of stay ≥ 6 h	2,626 (22.9)	1,665 (19.6)	961 (31.9)	< 0.0001

*NHI *national health insurance

### Sensitivity analysis of comparisons among the disposition subgroups

Table 2 shows the comparison of the characteristics of patients with SA-related ED visits in the discharge group. Except for the season of ED visit and time from event to arrival, most characteristics differed significantly among the subgroups. In the AMA subgroup, the proportion of female and late-adolescence patients was higher than that in the other discharge subgroups (73.0% vs. 57.5–54.1% in females and 68.2% vs. 60.8–63.5% in late-adolescence patients, respectively). In addition, the proportion of patients with a severe [1–3] KTAS score (64.4%), EMS use (49.7%), poisoning as the method of SA (50.0%), psychiatry as the major treatment subject (34.2%), hospital beds over 600 (56.1%), and > 6 h length of stay (29.1%) was significantly higher in the AMA subgroup than in other discharge subgroups.

**Table 2:** Comparison of characteristics of patients who attempted suicide based on discharge subgroups

Variable	Discharge (*n* = 8,456)	Discharged AMA (*n* = 3,231)	Discharged to home (*n* = 4,970 )	Others (*n* = 255)	*p* value
	n(%)	n(%)	n(%)	n(%)	
Age					
14–16 years	3,066 (36.3)	1,026 (31.7)	1,947 (39.1)	93 (36.4)	< 0.0001
17–19 years	5,390 (63.7)	2,205 (68.2)	3,023 (60.8)	162 (63.5)	
Female sex	5,357 (63.3)	2,360 (73.0)	2,859 (57.5)	138 (54.1)	< 0.0001
Insurance type					
NHI	6,840 (80.8)	2,693 (83.3)	3,959 (79.7)	188 (73.7)	< 0.0001
Medicaid	721 (8.5)	264 (8.2)	426 (8.6)	31 (12.1)	
Other	895 (10.5)	274 (8.5)	585 (11.7)	36 (14.1)	
EMS use	3,337 (39.4)	1,606 (49.7)	1,624 (32.6)	107 (41.9)	< 0.0001
KTAS score					
1–3	3,987 (47.1)	2,082 (64.4)	1,793 (36.0)	112 (43.9)	< 0.0001
4–5	4,469 (52.8)	1,149 (35.5)	3,177 (63.9)	143 (56.0)	
Year					
2016	1,344 (15.8)	447 (13.8)	820 (16.5)	77 (30.2)	< 0.0001
2017	1,677 (19.8)	579 (17.9)	1,036 (20.8)	62 (24.3)	
2018	2,514 (29.7)	986 (30.5)	1,480 (29.7)	48 (18.8)	
2019	2,921 (34.5)	1,219 (37.7)	1,634 (32.8)	68 (26.6)	
Season					
Spring	2,028 (24.0)	793 (24.5)	1,177 (23.6)	58 (22.7)	0.918
Summer	2,401 (28.4)	912 (28.2)	1,415 (28.4)	74 (29.0)	
Fall	2,320 (27.4)	890 (27.5)	1,356 (27.2)	74 (29.0)	
Winter	1,707 (20.2)	636 (19.6)	1,022 (20.5)	49 (19.2)	
Method of suicide attempts					
Poisoning	2,916 (34.5)	1,614 (50.0)	1,220 (24.6)	82 (32.1)	< 0.0001
Cutting and piercing	4,070 (48.2)	1,268 (39.3)	2,674 (53.8)	128 (50.2)	
Others	1,464 (17.3)	346 (10.7)	1,073 (21.6)	45 (17.6)	
Major treatment subject					
Psychiatry	1,715 (20.3)	1,105 (34.2)	591 (11.8)	19 (7.5)	< 0.0001
Time from event to arrival (h)	1.0 (0.6–3.0)	1.2 (0.7–3.3)	1.0 (0.5–2.9)	1.0 (0.5–2.3)	0.430
Time of arrival					
07:00–14:59	1,822 (21.5)	702( 21.7)	1,050 (21.1)	70 (27.4)	0.005
15:00–22:59	3,236 (38.2)	1,200 (37.1)	1,961 (39.4)	75 (29.4)	
23:00–06:59	3,398 (40.1)	1,329 (41.1)	1,959 (39.4)	110 (43.1)	
Region					
Urban	4,014 (47.5)	1,805 (55.8)	2,132 (42.9)	77 (30.2)	< 0.0001
Rural	4,442 (52.5)	1,426 (44.1)	2,838 (57.1)	178 (69.8)	
Hospital beds					
≥ 600	3,996 (47.2)	1,813 (56.1)	2,106 (42.3)	77 (30.2)	< 0.0001
300–599	1,050 (12.4)	163 (5.0)	824 (16.5)	63 (24.7)	
< 300	3,410 (40.3)	1,255 (38.8)	2,040 (41.0)	115 (45.1)	
Length of stay ≥ 6 h	1,665 (19.6)	943 (29.1)	675 (13.5)	47 (18.4)	< 0.0001

*NHI *national health insurance, *AMA *against medical advice

Table 3 shows the comparison of characteristics of patients with SA-related ED visits in the hospitalization group. Characteristics such as age group, insurance type, EMS use, KTAS score, method of SA, major treatment subject, number of hospital beds, and length of stay were significantly different between the GW and ICU admission subgroups. Overall, > 90% of patients in the ICU admission subgroup had a severe KTAS score. In addition, poisoning was the method of SA for 79.5% of patients in the ICU admission subgroup. Only 1.1% of patients in the ICU admission subgroup had psychiatry as the major treatment subject. The proportion of patients with a > 6 h length of stay in EDs was higher in the GW admission subgroup than in the ICU admission subgroup (38.9% vs. 18.8%).

**Table 3:** Characteristics of patients who attempted suicide based on hospital admission subgroups

Variable	Hospitalization (*n* = 3,006)	General ward admissions (*n* = 1,958)	Intensive care unit admissions (*n* = 1,048)	*p* value
	n(%)	n(%)	n(%)	
Age				
14–16 years	1,251 (41.6)	863 (44.1)	388 (37.0)	0.0002
17–19 years	1,755 (58.3)	1,095 (55.9)	660 (63.0)	
Female sex	2,119 (70.4)	1,364 (69.6)	755 (72.0)	0.172
Insurance type				
NHI	2,373 (78.9)	1,579 (80.6)	794 (75.8)	0.002
Medicaid	284 (9.45)	179 (9.1)	105 (10.0)	
Other	349 (11.6)	200 (10.2)	149 (14.2)	
EMS use	1,332 (44.3)	793 (40.5)	539 (51.4)	< 0.0001
KTAS score				
1–3	2,403 (79.9)	1,455 (74.3)	948 (90.4)	< 0.0001
4–5	603 (20.0)	503 (25.6)	100 (9.5)	
Year				
2016	550 (18.3)	361 (18.4)	189 (18.0)	0.115
2017	589 (19.5)	384 (19.6)	205 (19.5)	
2018	896 (29.8)	607 (31.0)	289 (27.5)	
2019	971 (32.3)	606 (31.0)	365 (34.8)	
Season				
Spring	732 (24.3)	484 (24.7)	248 (23.6)	0.273
Summer	862 (28.6)	579 (29.6)	283 (27.0)	
Fall	795 (26.4)	501 (25.6)	294 (28.0)	
Winter	617 (20.5)	394 (20.1)	223 (21.2)	
Method of suicide attempt				
Poisoning	2,093 (69.7)	1,260 (64.4)	833 (79.5)	< 0.0001
Cutting and piercing	437 (14.5)	410 (20.9)	27 (2.6)	
Others	473 (15.7)	286 (14.6)	187 (17.8)	
Major treatment subject				
Psychiatry	361 (12.0)	350 (17.9)	11 (1.1)	< 0.0001
Time from event to arrival (h)	2.0 (0.8–6.0)	2.0 (0.8–6.4)	2.0 (0.8–5.4)	
Time of arrival				
07:00–14:59	871 (28.9)	588 (30.0)	283 (27.0)	0.205
15:00–22:59	1,178 (39.1)	752 (38.4)	426 (40.7)	
23:00–06:59	957 (31.8)	618 (31.5)	339 (32.3)	
Region				
Urban	1,365 (45.4)	901 (46.0)	464 (44.3)	0.360
Rural	1,641 (54.5)	1,057 (54.0)	584 (55.7)	
Hospital beds				
≥ 600	1,512 (50.3)	1,051 (53.6)	461 (43.9)	< 0.0001
300–599	383 (12.7)	227 (11.5)	156 (14.8)	
< 300	1,111 (36.9)	680 (34.7)	431 (41.1)	
Length of stay ≥ 6 h	961 (31.9)	763 (38.9)	198 (18.8)	< 0.0001

*NHI *national health insurance

Figure S3 shows the comparison of the annual trends in SA-related ED visits from 2016 to 2019 based on the type of disposition, including the death, transfer, and discharge groups. In the death group, the proportion of patients pronounced dead on arrival decreased but that of patients who had an out of hospital cardiac arrest increased from 2016 to 2019. In the transfer group, the proportion of patients transferred due to insufficient beds increased but that due to requests from the patient or guardian decreased. In the discharge group, the proportion of patients who requested discharge AMA increased. Table S1 presents the top 10 most common diagnoses by the type of disposition. The most common diagnosis in the discharge group was open-wound injury, while poisoning or toxic effect was the most common in the GW and ICU admission subgroups.

## Discussion

To the best of our knowledge, this study is the first nationwide analysis of trends in SA-related ED visits among adolescents in Korea. This study examined the incidence of SA-related ED visits among adolescents in South Korea from 2016 to 2019 and compared the demographic and clinical characteristics of patients based on the dispositions of these SA-related ED visits. Using joinpoint regression analysis, we found a steep increase in the incidence rates of SA-related ED visits among female and mid-adolescent (aged 14–16 years) patients compared with that noted among male and late-adolescent patients (aged 17–19 years). Moreover, the distinct difference depending on the disposition type for the characteristics of patients with SA-related ED visits were significant in terms of age, sex, EMS use, the severe KTAS score, the method of SAs, time from event to arrival, and hospital beds, and length of stay in ED.

In this study, we found that the incidence rates of SA-related ED visits was increasing in female adolescents from 2016 to 2019. This finding is consistent with results reported in previous studies conducted in Korea about the increase in number of female teenagers engaging in suicidal behaviors, including SA as well as suicidal ideation and suicidal planning [6, 29]. The increase in SAs among female seems to contribute to the stagnation of the suicide rate despite the introduction of suicide prevention programs in Korea. Similar data have been reported in other developed countries. A US study showed that the prevalence of suicidal ideation in females increased from 2009 to 2019 (APC: 4.0%; 95% CI: 2.5–5.6%) but not in males (APC: 1.4%; 95% CI: −1.2–4.0%) [30]. Even considering the prevalence of suicide risk factors such as depression varies by region [31–33], our findings have important implications for suicide prevention efforts in countries where the decline in suicide rates has stagnated.

Female adolescents have been reported to be more sensitive to parental socio-economic status than males [34]. It is well-known that female adolescence is associated with high stress levels due to significant changes regarding physical, psychological, and social aspects [35]. Moreover, female adolescents from poor families are more likely to join the labor market at an early age and to forgo college admission than male adolescents in Korea according to a National Youth Policy report [34]. This suggests that a more careful gender-sensitive approach is needed for effective interventions regarding self-injury behaviors among adolescents from poor families. Furthermore, female adolescents’ unique upward trend for SAs seems to reflect the trajectory of depression-related symptoms; in fact, the incidence of depression-related symptoms has been increasing only among women since 2005 [36–38]. Xiao et al. suggested that females are more vulnerable to school bullying, cyberbullying, and peer sacrifice than male adolescents and that female-specific preventive programs are therefore needed for suicide intervention [30, 39–41]. Actually, recent cross-sectional studies of Korean adolescents showed that female adolescents had longer Internet usage (e.g., online social networking services) time than male adolescents, and Internet usage time was associated with increased stress levels and suicidal ideation [42, 43]. Our results for the sex-difference also support these findings of previous studies [44, 45], and suggesting the need to consider both individual stress-diathesis risks and societal-contextual factors at large [30]. Moreover, a Korean study reported that the hospitalization rate for SAs among patients diagnosed with a psychiatric illness, such as depression, bipolar disorder, post-traumatic stress disorder, anxiety disorder, or somatoform disorder, from 2008 to 2013 was higher among females than males [46]. Although there has been a decrease in suicide rates after the Act for Prevention of Suicide and the Creation of Culture of Respect for Life was implemented in 2011 (from 31.7 to 27.3 deaths per 100,000 people) [47], the rate of SAs among female adolescents was still found to be increased in our study. Thus, our findings further highlight the clinical significance of establishing prevention strategies for adolescent suicides for females.

We also found that approximately two-thirds of adolescents with SA-related visits are discharged from the hospital to home. The discharge AMA is common among psychiatric inpatients, but it has received little attention in medical research over the last decade [48]. Based on previous research, patients who requested discharge AMA have poor long-term prognosis, high re-hospitalization rate, and low outpatient service use. Nevertheless, a few studies have reported on suicide mortality among patients who opt for discharge AMA [45, 49–51]. In a large cohort of psychiatric inpatients, Kuo et al. reported that discharge AMA was associated with a significantly increased risk of suicide mortality. Their findings provide valuable insight for clinicians making decisions regarding discharge and post-discharge management of patients discharged AMA [48]. Our finding which has shown an annual increased number of discharge group from adolescents with SA-related ED visits, suggested that this group should be more considered when suicide prevention programs designed.

The present study has some limitations. First, no patient data regarding single or multiple SAs were available. Second, the results regarding characteristics based on type of disposition are based exclusively on data such as those obtained from the death and transfer groups. Third, this study is the absence of data regarding a community or non-ED comparator group. So this may be limited the generalizability of the findings among suicide attempters, as characteristics such as the rate of the method for suicide attempt in these groups may differ. However, we tried to compare to the method of suicide attempt by the type of disposition between mild and severe group in ED visits. Still, more consideration for non-ED visitors or community group should be needed. Fourth, patient data regarding several characteristics, including socio-economic status and SA-related family history or environment, were not available. Therefore, future epidemiological research is warranted, considering factors such as being out of school as well as social and interpersonal problems (including family crises and relationships with parents), to investigate the risks associated with SAs among adolescents.

Nevertheless, our study has several strengths. First, this is the first nationwide analysis of SA-related ED visits among adolescents in Korea. Second, this study included those NEDIS variables that were identified at the time of ED visits along with medical information such as the KTAS score and EMS use, so it may be sufficient to predict the in-hospital mortality risk during the early stages of ED visits. It should be considered that it was impossible to accurately determine the SA rates in this study, since SA data were based mainly on ED visits, and thus, individuals who visited general practitioners or who did not seek any medical assistance, who are estimated to be account for 50–80% of all those who attempt suicide, could not be included. Therefore, it could be concluded that the exact incidence rates of SAs are much higher than those presented in this study. This is an evidence-based study aiming at supporting disposition decisions to minimize unnecessary admissions of low-risk patients and maximize observation of high-risk patients, irrespective of diagnoses.

## Conclusions

We found that the population rates of SA-related ED visits among female and mid-adolescent (aged 14–16 years) patients were rapidly increased compared with that noted among male and late-adolescent patients. The distinct difference in the characteristics of patients with SA-related ED visits were significant depending on the type of disposition. The findings of this study have significant implications for planning of interventions to prevent SAs among adolescents. Identification of the distinct characteristics of patients who visited an ED has clinical significance in terms of facilitating the establishment of prevention strategies for adolescent suicides. Our findings also suggest that more funding support and policy advocacy are warranted for developing more comprehensive and culturally appropriate suicide prevention programs targeting different risks of ideation and attempts across sex and age groups.

## Supplementary Information

**Additional file 1.**

## Data Availability

The datasets used and/or analyzed during the present study are available from the corresponding author on reasonable request.

## Abbreviations

SA: suicide attempt
ED: emergency department
NEDIS: National Emergency Department Information System
APC: annual percentage change
EMS: emergency medical service
KTAS: Korean Triage and Acuity Scale
ICD-10: International Classification of Disease 10th Edition
AMA: against medical advice
GW: general ward
ICU: intensive care unit
