# Supplementary material for: Suicide attempt-related emergency department visits among adolescents: a nationwide population-based study in Korea, 2016–2019
Source: BMC Psychiatry. 2022 Jun 22;22:418. doi: 10.1186/s12888-022-04043-6 (PMC9215032; doi:10.1186/s12888-022-04043-6)
Supplement: Supplementary file 1 — Additional file 1. [file 12888_2022_4043_MOESM1_ESM.docx]

**Supplemental material**

Figure S1. Trend in the age- and sex- standardized population rate of suicide attempt-related ED visits from 2016 to 2019


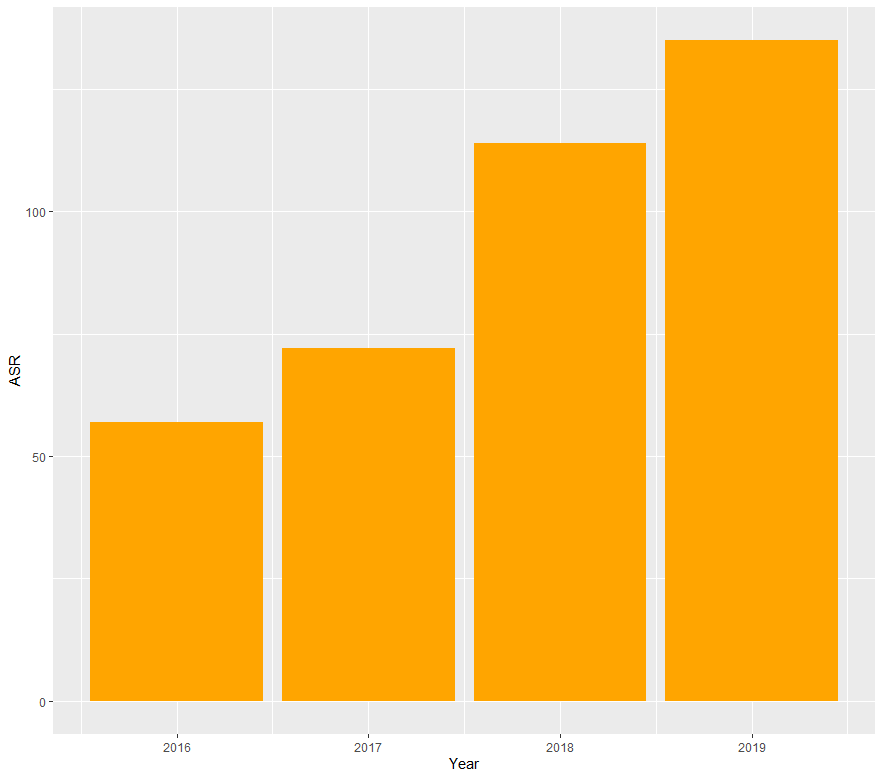


Note: ASR, age- and sex-standardized incidence rates of suicide attempt-related ED visits per 100,000 persons (with the 2020 census population as the standard population).

Figure S2. Trends in suicide attempt-related ED visits among adolescents based on disposition

| 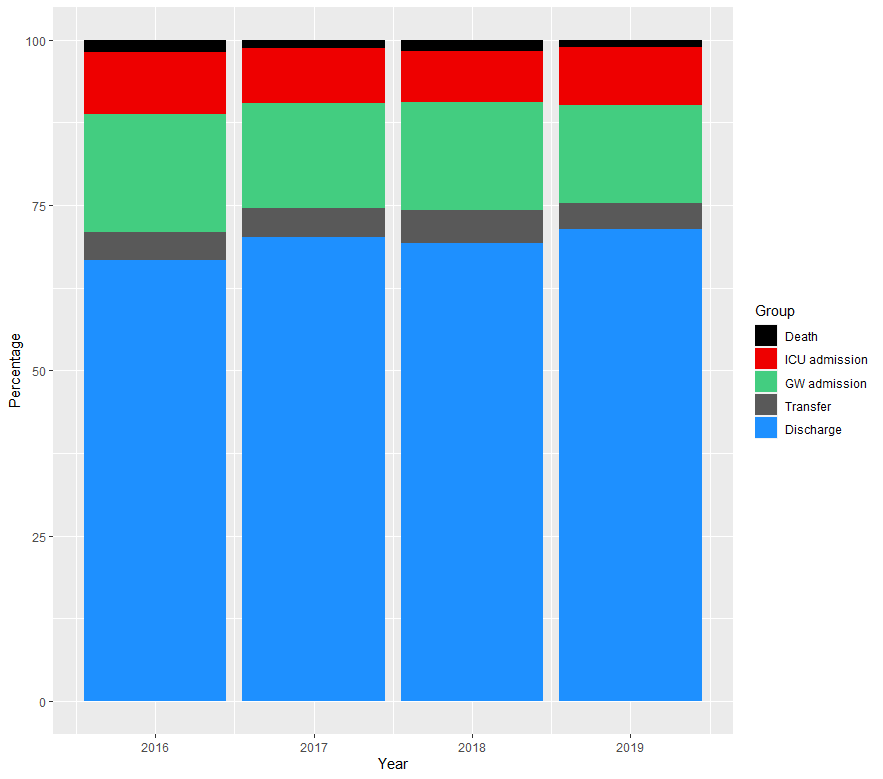  Note: ED, emergency department; ICU, intensive care unit; GW, general ward |
| --- |

Figure S3. Detailed trends in suicide attempt-related ED visits among adolescents based on disposition

| 1. Death group | 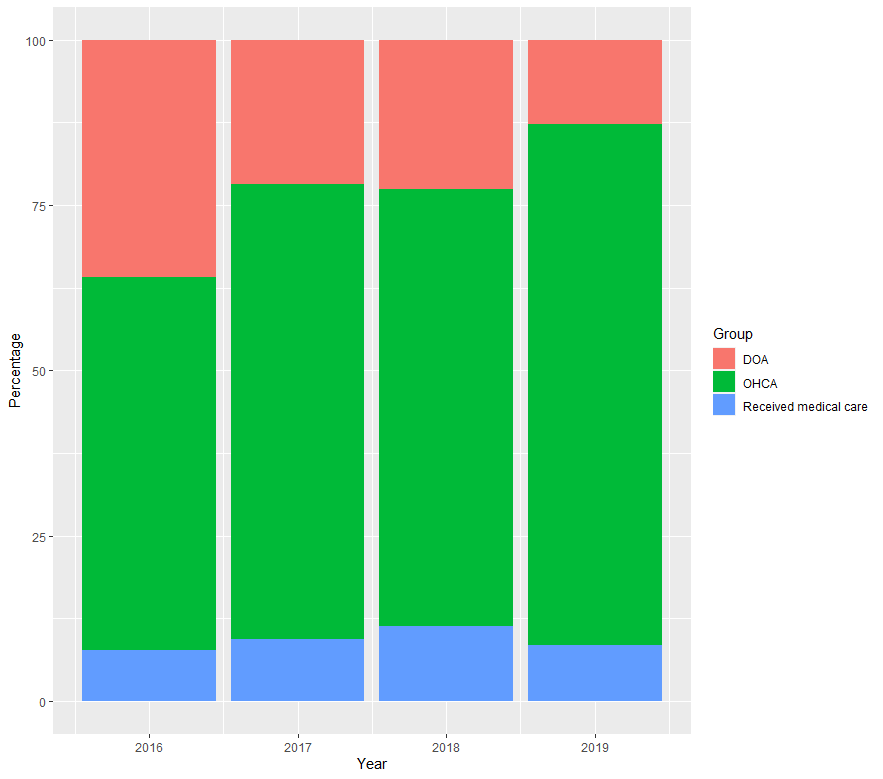 |
| --- | --- |
| (b) Transfer group | 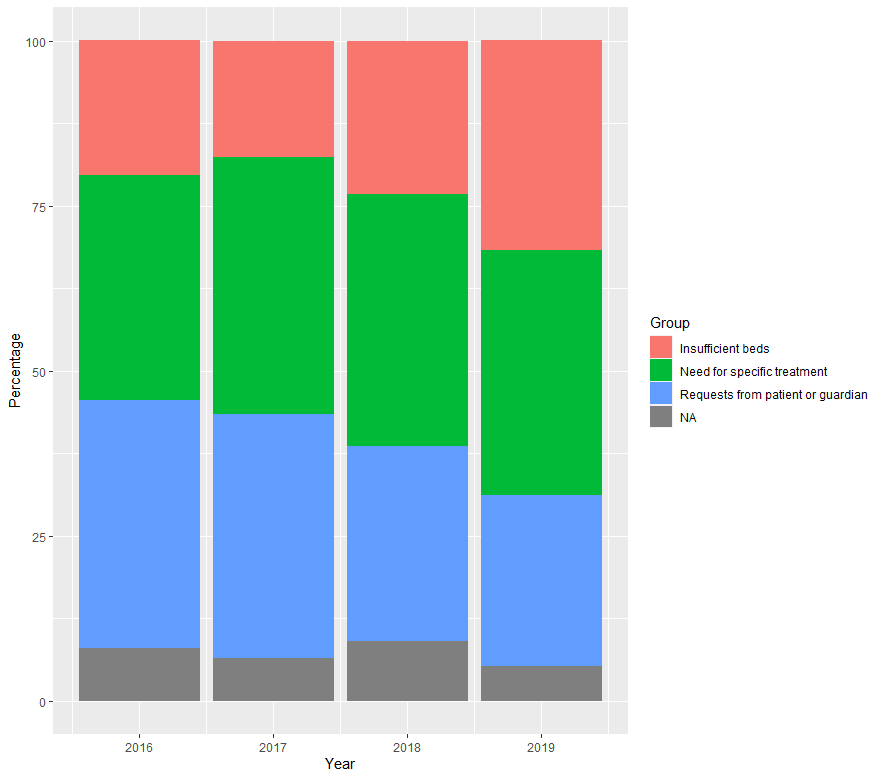 |
| (c) Discharge group | 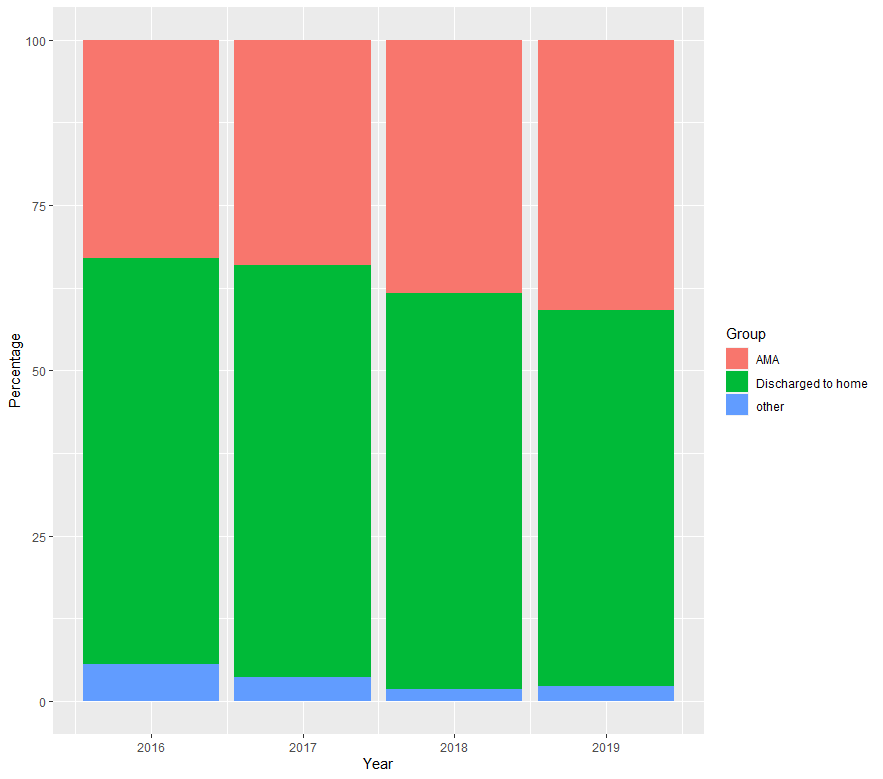 |

Note: DOA, dead on arrival; OHCA, out of hospital cardiac arrest; NA, others; AMA, against medical advice.

Table S1. The ten most common diagnoses by disposition

| No. | Total  (n=11,462) | | Discharge group  (n=8,456) | | General ward admissions subgroup  (n=1,958) | | Intensive care unit admissions subgroup (n=1,048) | |
| --- | --- | --- | --- | --- | --- | --- | --- | --- |
| 1 | S61 | 1,994 (17.3) | S61 | 1,855 (21.9) | T50 | 364 (18.5) | T50 | 276 (26.3) |
| 2 | T50 | 1,373 (11.9) | S51 | 787 (9.3) | T39 | 272 (13.8) | T42 | 100 (9.5) |
| 3 | S51 | 845 (7.3) | T50 | 733 (8.6) | S61 | 135 (6.8) | T39 | 97 (9.2) |
| 4 | T39 | 604 (5.2) | F32 | 438 (5.1) | F32 | 113 (5.7) | T65 | 89 (8.4) |
| 5 | F32 | 558 (4.8) | S60 | 433 (5.1) | T42 | 108 (5.5) | F19 | 69 (6.5) |
| 6 | T42 | 501 (4.3) | T42 | 293 (3.4) | F19 | 90 (4.5) | T43 | 59 (5.6) |
| 7 | F19 | 441 (3.8) | F19 | 282 (3.3) | T65 | 87 (4.4) | S32 | 30 (2.8) |
| 8 | S60 | 441 (3.8) | R45 | 270 (3.1) | S66 | 60 (3.0) | F13 | 21 (2.0) |
| 9 | T65 | 382 (3.3) | T39 | 235 (2.7) | S51 | 57 (2.9) | T58 | 20 (1.9) |
| 10 | R45 | 301 (2.6) | Z91 | 230 (2.7) | T43 | 57 (2.9) | I46 | 18 (1.7) |

| Note: S61, Open wound of wrist and hand; T50, Poisoning by diuretics or other unspecified drugs; S51, Open wound of forearm; T39, Poisoning by non-opioid analgesics; F32, Depressive episode; T42, Poisoning by antiepileptic; F19, Misuse of drugs NOS; S60, Superficial injury of wrist and hand; T65, Toxic effect of other and unspecified substances; R45, Symptoms and signs involving emotional state; Z91, Personal history of risk factors; S66, Injury of muscle and tendon at wrist and hand level; T43, Poisoning by psychotropic drugs; S32, Fracture of lumbar spine and pelvis; F13, Mental and behavioral disorders due to use of sedatives or hypnotics; T58, Toxic effect of carbon monoxide; I46, Cardiac arrest |
| --- |
|  |
|  |
|  |
